# The polycaprolactone/silk fibroin/carbonate hydroxyapatite electrospun scaffold promotes bone reconstruction by regulating the polarization of macrophages

**DOI:** 10.1093/rb/rbac035

**Published:** 2022-06-11

**Authors:** Xiaoshi Jia, Jing Zhou, Jinqiu Ning, Maoquan Li, Yitong Yao, Xiaodong Wang, Yutao Jian, Ke Zhao

**Affiliations:** Department of Prosthodontics, Guanghua School of Stomatology, Hospital of Stomatology, Sun Yat-sen University, Guangzhou, Guangdong 510055, China; Guangdong Provincial Key Laboratory of Stomatology, Guangzhou, Guangdong 510055, China; Department of Prosthodontics, Guanghua School of Stomatology, Hospital of Stomatology, Sun Yat-sen University, Guangzhou, Guangdong 510055, China; Guangdong Provincial Key Laboratory of Stomatology, Guangzhou, Guangdong 510055, China; Department of Prosthodontics, Guanghua School of Stomatology, Hospital of Stomatology, Sun Yat-sen University, Guangzhou, Guangdong 510055, China; Guangdong Provincial Key Laboratory of Stomatology, Guangzhou, Guangdong 510055, China; Department of Prosthodontics, Guanghua School of Stomatology, Hospital of Stomatology, Sun Yat-sen University, Guangzhou, Guangdong 510055, China; Guangdong Provincial Key Laboratory of Stomatology, Guangzhou, Guangdong 510055, China; Department of Prosthodontics, Guanghua School of Stomatology, Hospital of Stomatology, Sun Yat-sen University, Guangzhou, Guangdong 510055, China; Guangdong Provincial Key Laboratory of Stomatology, Guangzhou, Guangdong 510055, China; Department of Prosthodontics, Guanghua School of Stomatology, Hospital of Stomatology, Sun Yat-sen University, Guangzhou, Guangdong 510055, China; Guangdong Provincial Key Laboratory of Stomatology, Guangzhou, Guangdong 510055, China; Department of Prosthodontics, Guanghua School of Stomatology, Hospital of Stomatology, Sun Yat-sen University, Guangzhou, Guangdong 510055, China; Guangdong Provincial Key Laboratory of Stomatology, Guangzhou, Guangdong 510055, China; Department of Prosthodontics, Guanghua School of Stomatology, Hospital of Stomatology, Sun Yat-sen University, Guangzhou, Guangdong 510055, China; Guangdong Provincial Key Laboratory of Stomatology, Guangzhou, Guangdong 510055, China

**Keywords:** bone reconstruction, PCL/SF/CHA scaffold, macrophage polarization, electrospinning

## Abstract

Macrophages are known to modulate the osteogenic environment of bone regeneration elicited by biological bone grafts. Alteration in certain chemical components tends to affect macrophages polarization. Comparatively to hydroxyapatite (HAp), carbonate hydroxyapatite (CHA) consists of 7.4 (wt%) carbonate ions and more closely resembles the mineral content of bone. It remains unknown whether CHA scaffolds or HA scaffolds have better osteogenic properties. In this study, we fabricated PCL/SF scaffold, PCL/SF/HAp scaffold and PCL/SF/CHA scaffold using the electrospinning technique. Despite comparable mechanical properties, the PCL/SF/CHA scaffold exhibited better osteogenic properties than the PCL/SF/HAp scaffold. Although no significant differences were observed between the two scaffolds for promoting osteoblast differentiation *in vitro*, the PCL/SF/CHA group appeared to be more effective at promoting bone regeneration in cranial defects *in vivo*. The PCL/SF/CHA scaffold was found to promote macrophage polarization toward M2 via activating the JAK/STAT5 pathway which caused a pro-osteogenic microenvironment to facilitate osteoblast differentiation. The results of this study indicated a higher potential of CHA to substitute HAp in the production of bone scaffolds for better bone regeneration.

## Graphical Abstract

**Figure:**
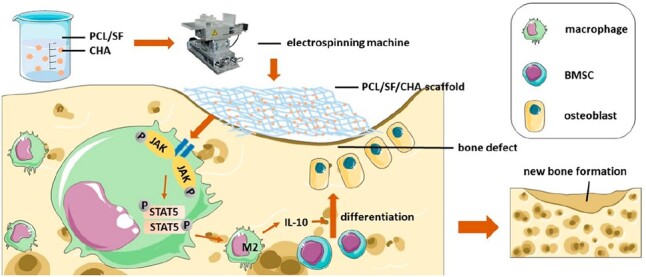

## Introduction

Bone defects resulting from chronic disease or trauma pose a substantial challenge for clinical treatment [1]. Bone-grafting materials are now recognized as an indispensable part of the armamentarium for the treatment as well as repair of bone defects [2]. The scaffolds should meet basic requirements such as sufficient mechanical properties, biocompatibility and biodegradability to facilitate better osteogenesis in the bone defects area. Currently, most osteoimmunomodulatory strategies attempt to enable the biomaterials to modulate the local environment from pro-inflammatory to be in favor of pro-regeneration. A significant role of macrophages phenotypes in the bone regeneration induced by biomaterials had been well reported and applied to many modification strategies [3].

As a simple and effective technique, electrospinning has been applied to prepare scaffolds with nanofibers that mimic the natural bone extracellular matrix [4]. The high surface-area-to-volume ratio of the scaffolds prepared using the electrospray technique aid in improving cell attachment [5]. Both SF and PCL are Food & Drug Administration-approved biomaterials that can be used in electrospinning as they are inexpensive and possess desirable biocompatibility. PCL/SF scaffolds have shown increased mechanical strength and improved biological properties as compared with pure SF scaffolds and pure PCL scaffolds respectively [4, 6].

Previous studies suggest that hydroxyapatite (Hap), which is one of the most widely used calcium phosphate (CaP) ceramics for the regeneration of bone tissue, can bind the aspartic acid or glutamic acid of osteocalcin (OCN) [7, 8]. Nanocrystalline HAp promotes osteoblast adhesion, differentiation and proliferation [9]. Nano carbonate hydroxyapatite (nCHA) is a kind of non-stoichiometric nano HAp (nHAp) with phosphate or hydroxide ions partially replaced by carbonate ions [10]. As compared to nHAp, nCHA consists of 7.4 (wt%) carbonate ions and more closely resembles the mineral portion of bone [11]. Recently, nCHA has gained increasing attention as a possible substitute for nHAp in the process of supporting bone tissue regeneration. To accurately mimic the carbonate-containing nature of HAp in bone, carbonate substituents have been incorporated into synthetic bioinspired nCHA. As compared to nHAp, nCHA not only has preferable biosorption resulting in improved bone remodeling [12] but it also reduces the secretion of inflammatory cytokines by macrophages [13]. However, it is still unknown whether the addition of carbonate hydroxyapatite (CHA) can improve the immunoregulatory performance of the scaffold without compromising other functions.

It has been found that macrophages regulate the osteogenic environment of biological bone grafts during the bone repair process [14, 15]. In response to stimulation by the microenvironment, macrophages can polarize into either M1 (pro-inflammation) or the M2 (anti-inflammation and tissue repair) phenotype [16]. It is generally accepted that M1 macrophages mainly produce pro-inflammatory cytokines such as IL-1β, TNF-α and IL-6 to reduce osteogenesis, whereas M2 macrophages secrete anti-inflammatory cytokines and growth factors to promote bone repair [17]. The plasticity of macrophages has made them an ideal target in bone repair research. A major focus of current research on bone repair is to develop bone graft scaffolds that can regulate the polarization of macrophages to establish a pro-osteogenic microenvironment for bone formation.

In this study, we have prepared PCL/SF/CHA scaffolds with PCL/SF and PCL/SF/HAp scaffolds being served as control groups. We hypothesized that PCL/SF/CHA scaffolds could regulate M2-type macrophages polarization to achieve a microenvironment conductive to osteogenesis. The objective of this study was to detect the mechanical, osteogenic and immunoregulatory properties of PCL/SF/CHA scaffolds and then explore its molecular mechanisms leading to the bone regeneration.

## Materials and methods

### Preparation of electrospun hybrid scaffolds

The scaffolds were prepared using the electrospinning technique as described [4], with some modifications. To yield the electrospinning solutions of PCL/SF, PCL and SF were dissolved in hexafluoroisopropanol at a weight ratio of 8:1 where the final concentration of SF was 2% (w/v). Separate solutions of PCL/SF/HAp and PCL/SF/CHA are prepared by addition of HAp and CHA powder, respectively, to the PCL/SF solution at a concentration of 2% (w/v) followed by overnight magnetically stirred at room temperature subsequently sonication for 3 min.

The electrospinning solutions were then transferred to a syringe with a blunt-end 22-gauge needle and electrospun (6 mL/h, 20 kV) from the spinneret tip to the grounded aluminum foil at the distance of 15 cm. Samples were subsequently dried overnight in a vacuum oven and immersed in 75% ethanol for 30 min before being stored in a desiccator. The final scaffolds thickness was 100 μm, weight/area ratio was 0.09 mg/cm^2^ and the content of HAp or CHA is 10%.

### Characterization of the scaffolds

The surface morphology of the electrospun scaffolds was analyzed with a SEM (PhenomProX, Phenom-World, Netherlands) at an accelerating voltage of 10 kV. Twenty fibers of each scaffold were selected randomly, and the diameters were measured with ImageJ software (NIH, Bethesda, MD, USA). The hydrophilicity of the scaffolds was determined by contact angle measurements using a contact angle apparatus (Dataphysics, Filderstadt, Germany).

### Mechanical properties of the hybrid scaffolds

CMT6103 universal testing machine (SANS, Guangdong, China) was used to test the scaffolds according to ASTM D882 [18]. Each sample was 10 mm in width and 50 mm in length. The test was performed at a speed of 50 mm/min^−1^ at room temperature with a load cell capacity of 50 N. Young’s modulus, tensile strength and maximum strain were measured.

### Degradation properties

Lipase from *Candida antarctica* lipase B (Novozymes, China) with a specific activity of 5 U/mg and a density of 1.20 g/mL was dissolved in phosphate-buffered saline (PBS, pH 7.4) to prepare an enzyme solution (30 U mL^−1^) [19]. The scaffolds were cut into squares (2.0 cm × 2.0 cm) and immersed in cell culture dishes (with diameters of 35 mm) containing 3 mL enzyme solution at 37°C. Specimens in each group were removed and lyophilized after a period of 2, 4, 6, 8, 10, 12 and 14 days. Each specimen was weighed before (W_0_) and after (W_t_) degradation as well as examined by SEM. Meanwhile, the pH of incubation medium solutions was tested by an electronic pH detector (SevenExcellence, METTLER TOLEDO, Switzerland).

### Cell culture

Bone marrow mesenchymal stem cells (BMSCs) were isolated from the femurs of 8-week-old C57BL/6J mice as previously described [20]. For this, bone marrow was flushed out and centrifuged. The cells were resuspended in α-MEM (HyClone) supplemented with 10% fetal bovine serum (FBS; Gibco, Life Technologies Corporation), 100 U/mL penicillin G and 100 mg/mL streptomycin (HyClone). After 48 h of incubation, non-adherent cells were removed while the adherent BMSCs were utilized in this study after three passages. BMSCs were seeded into 24-well plates with sterilized scaffolds (d = 1.5 cm, w = 0.16 mg) at a density of 5 × 10^4^ cells/cm^2^ in growth medium. At 70–90% confluence of BMSCs, the growth medium was replaced with osteogenic-inducing medium and changed every other day for 14 or 21 days.

RAW 264.7 cells were incubated in DMEM supplemented with 10% FBS, 100 U/mL penicillin G and 100 mg/mL streptomycin at 37°C in a humidified atmosphere containing 5% CO_2_ and seeded into six-well plates with sterilized scaffolds (d = 3.4 cm, w = 0.83 mg) at a density of 1 × 10^6^ cells/cm^2^.

### IF staining of cells on the scaffolds

BMSCs were fixed with 4% (g/mL) paraformaldehyde for 15 min. After being washed three times in PBS, the cells were stained by FITC-labeled phalloidin (Beyotime, China) and DAPI (Beyotime, China). BMSCs morphology was observed by confocal laser scanning microscopy (LSM800, Zeiss, Jena, Germany). The experiment was performed in triplicate.

RAW 264.7 cells cultured on the scaffolds were fixed with 4% (g/mL) paraformaldehyde for 15 min. After being washed three times in PBS, the cells were permeabilized with 1% Triton X-100 for 30 min and blocked with 0.1% bovine serum albumin (BSA) for 1 h. The cells were incubated with primary antibodies against iNOS (1:100, Abcam, ab15323) and CD206 (1:400, Abcam, ab64693) overnight at 4°C. Then cells were rinsed three times with PBS and incubated with the corresponding secondary antibodies (Alexa Fluor^®^ 488- or 594-conjugated goat anti-rabbit or anti-mouse IgG; both 1:1000; both Abcam; cat. nos. ab150113 and ab150080, respectively). After rinsed three times with PBS, the cells were stained with 10 µg/mL DAPI for 5 min at 25°C. Images were captured using a laser scanning confocal microscope (×400 magnification; Zeiss GmbH; cat. no. LSM780). All experiments were performed in triplicate.

### Proliferation of BMSCs on electrospun nanofibrous scaffolds

The proliferation of cells was analyzed by using the Cell Counting Kit-8 (CCK-8) assay (Beyotime Biotech, China) according to the manufacturer’s instructions. BMSCs and RAW 264.7 cells (4 × 10^3^) were seeded into the 48-well plate with sterilized scaffolds (d = 0.9 cm, w = 0.057 mg). The CCK-8 solution was added to each well of BMSCs after 1, 4 and 7 days, and to the wells of RAW 264.7 cells after 6, 24 and 48 h. After incubation at 37°C for 120 min, the absorbance value of the supernatant was measured at 450 nm by a microplate spectrophotometer (Epoch 2, BioTek, USA). The experiment was performed in triplicate.

### Subcutaneous implantation of scaffolds

A total of 15 mature female Sprague-Dawley (SD) rats (8-week-old, mean body weight 200 g) were purchased from Sun Yat-Sen University and divided into three groups: a PCL/SF group, a PCL/SF/HAP group and a PCL/SF/CHA group. All animal experiments were approved by the Institutional Animal Care and Use Committee, Sun Yat-Sen University. The surgical procedures were performed following the *Guide for the Care and Use of Laboratory Animals* published by the National Academy of Sciences. The SD rats were anesthetized by 1% pentobarbital sodium (40 mg/kg, ip). The skin incisions were made longitudinally by sharp dissection beside the midline of the back. To form a pouch, the subcutaneous tissues were separated bluntly. A scaffold was implanted into each pouch based on the group. The wounds were carefully sutured. The rats were euthanized with sodium pentobarbital (200 mg/kg, ip) after 4 weeks.

### Preparation of critical-sized calvarial bone defect models

A total of 60 mature female SD rats (8-week-old, mean body weight 200 g) were purchased from Sun Yat-Sen University and divided into four groups (*n* = 5): a blank group without implanted materials, a PCL/SF group, a PCL/SF/HAp group and a PCL/SF/CHA group. All animal experiments were approved by the Institutional Animal Care and Use Committee, Sun Yat-Sen University. The surgical procedures were performed following the *Guide for the Care and Use of Laboratory Animals* published by the National Academy of Sciences. The SD rats were anesthetized by 1% pentobarbital sodium (40 mg/kg, ip). The critical-sized calvarial bone defects (CSDs) were created on the epicranium of the SD rats using an 8 mm trephine. The prepared scaffolds were filled and fixed in the CSD circle of corresponding groups. The wounds were then carefully sutured. The rats were euthanized after 4, 8 or 12 weeks with sodium pentobarbital (200 mg/kg, ip).

### Micro-CT analysis

The calvarial bone samples were excised and fixed in 4% (w/v) paraformaldehyde for 24 h. The new bone formation within the defect region was assessed using a micro-CT system (SCANCO μCT 100, Scanco Medical AG, Switzerland). The samples were scanned with X-ray beam energy of 70 kV, beam intensity of 200 mA and spatial resolution of 30 μm. A 5-mm thick aluminum sheet was used as filter. The threshold level was from 220 to 1000. An area of defective bone was determined using the 2D pseudo-color image after reconstructing the 3D morphology of the calvarial bone, in which HU values were presented using a gradient color scheme. Based on the difference in bone density between the new bone and the surrounding skull, areas with lower bone density were delineated.

### Histological analysis

Following micro-CT scanning, the samples were decalcified in 0.5 M EDTA at pH 7.4, before being gradient-dehydrated and embedded in paraffin. Serial sections of 5 μm were cut and mounted on polylysine-coated slides. Deparaffinized and rehydrated sections were then stained with hematoxylin and eosin and Masson’s trichrome. Another set of sections were incubated with 0.3% hydrogen peroxide (0.3 mL H_2_O_2_ diluted in 99.7 mL methyl alcohol) for 20 min, followed by 5% (w/v) BSA (Beyotime, China). Afterward, the sections were incubated with primary antibodies against Col I (1:200 in PBS, SANTA, sc-59772), Runx2 (1:200 in PBS, Abcam, ab192256), iNOS (1:400 in PBS, Abcam, ab15323) or CD206 (1:400 in PBS, Abcam, ab64693) overnight at 4°C, by the manufacturer’s protocol. The sections were incubated using an UltraSensitive SP immunohistochemistry kit (Fuzhou Maixin Biotechnology, Ltd., China) and visualized by 3,3-diaminobenzidine tetrahydrochloride (Fuzhou Maixin Biotechnology, Ltd., China). Finally, the sections were counterstained with hematoxylin. Images of the slices were obtained with a digital slice scanner (Aperio AT2, Leica Biosystems, Germany). Image J software was used to quantify the ratio of bone formation area and positive stained cells.

### RNA extraction and RT-qPCR

Total RNA was extracted using an RNA extraction kit (RN001, ESscience, China) according to the manufacturer’s instructions. Nanodrop2000 (Thermo Fisher Scientific Inc.) was used to measure the concentration and quality of the total RNA in samples. Complementary DNA was synthesized from 1 μg of total RNA by using the PrimeScript RT-PCR kit (RR047A, TaKaRa, Japan) and instructions provided in the manufacturer’s protocol. RT-qPCR was performed with a PrimeScript RT-PCR kit (RR820A, TaKaRa, Japan). The primers used in this study are listed in Table 1. The experiment was performed in triplicate.

**Table 1.** List of primer sequences used in the qRT-PCR

Genes	Species	Forward（5′-3′）	Reverse（5′-3′）
GAPDH	Rat	AGTGCCAGCCTCGTCTCATA	GGGTTTCCCGTTGATGACCA
Col-1	Rat	GTATTGCTGGTGCTCTGGGT	GGACCAATGTTGCCAGGGTA
Alp	Rat	TGCAGGATCGGAACGTCAAT	GAGTTGGTAAGGCAGGGTCC
Osx	Rat	GCATCTGAAAGCCCACTTGC	AGTGGTCGCTTCGGGTAAAG
Ocn	Rat	TTGTGACGAGCTAGCGGAC	CCACCACAATGGACAGACTCG
GAPDH	Mouse	CTCCCACTCTTCCACCTTCG	TTGCTGTAGCCGTATTCATT
CD206	Mouse	TTCAGCTATTGGACGCGAGG	GAATCTGACACCCAGCGGAA
iNOS	Mouse	GCTCGCTTTGCCACGGACGA	AAGGCAGCGGGCACATGCAA
IL-10	Mouse	CTGCTCCACTGCCTTGCTCTTATT	GTGAAGACTTTCTTTCAAACAAAG
IL-1β	Mouse	TGCCACCTTTTGACAGTGATG	TGATGTGCTGCTGCGAGATT

### Alkaline phosphatase (ALP) activity of BMSCs

After osteogenic differentiation, BMSCs were detected by using an Alkaline Phosphatase Assay Kit (C3206, Beyotime Biotech, China) as per the manufacturer’s instructions. The ALP activity was measured by a microplate spectrophotometer (Epoch 2, BioTek, USA) at 405 nm. The experiment was performed in triplicate.

### ELISA assay

The supernatant of RAW 264.7 cells grown on scaffolds was collected. The quantities of IL-10 and IL-1β in the supernatant were measured with mouse ELISA kits (CUSABIO TECHNOLOGY LLC, CA) according to the manufacturer’s instructions. A microplate spectrophotometer (Epoch 2, BioTek, USA) was used to measure the absorbance at 450 nm. The experiment was performed in triplicate.

### Flow cytometry

After being cultured on the scaffolds, RAW 264.7 cells were resuspended and fixed with 100% methanol for 5 min, followed by incubation in 0.5% (v/v) Triton-X100/PBS for 1 h and in 1% (w/v) BSA for 30 min. Initially, the mixed solutions were incubated in primary antibody against iNOS (1:2000, Abcam, ab15323) at 4°C for 2 h followed by secondary antibody (1:2000, Abcam, ab150077) at 22°C for 2 h in the dark. For the test of CD206, RAW 264.7 cells were fixed and permeabilized with the Intracellular Fixation and Permeabilization Buffer Set (cat. 88-8824, eBioscience™) according to the manufacturer protocols. After that, the cells were incubated in antibody (cat. 12-2061-80, eBioscience™) at 4°C for 2 h in the dark. Acquisition of >10 000 events was performed by means of a flow cytometer (CytoFLEX S, Beckman Coulter, USA). The experiment was performed in triplicate.

### Protein extraction and western blotting

RAW 264.7 cells were collected with RIPA buffer. The protein concentration was measured using a bicinchoninic acid assay kit (Thermo Scientific, Rockford, USA). Afterward, the proteins were mixed with loading buffer and heated at 95°C for 10 min for denaturation.

SDS-PAGE was used to separate the proteins, which were then transferred to nitrocellulose membranes. The membranes were blocked by 5% skim milk (g/mL) or 5% BSA (g/mL) for 60 min at room temperature and then incubated with primary antibodies against p-AKT (1:2000, CST, 9271S), AKT (1:1000, CST, 4691 T), p-p65 (1:1000, CST, 3033 T), p65 (1:1000, CST, 8242 T), β-actin (1:10000, CST, 58169S), STAT5 (1:1000, CST, 25656S), p-STAT5 (1:1000, CST, 9314S), p-JAK1 (1:1000, CST, 74129 T), p-JAK2 (1:1000, CST, 4406 T) and p-JAK3 (1:1000, CST, 5031 T) at 4°C overnight, followed by secondary antibodies for 1 h at room temperature. The primary and secondary antibodies were all diluted in 5% (g/mL) BSA. Protein bands on the membranes were visualized using a Western Bright ECL HRP substrate kit (Advansta, USA). Image J software was used to quantify the gray value of each belt. The tests were repeated in triplicate.

### Statistical analysis

All *in vitro* experiments were performed in triplicate. The results were presented as means ± SD and analyzed with SPSS 25.0 software (IMB, USA) and GraphPad Prism software (GraphPad, San Diego, CA, USA). Data were analyzed by one-way analysis of variance followed by a Student–Newman–Keuls *post hoc* test for pair-wise comparisons. A *P-*values < 0.05 were considered statistically significant.

## Results

### Physical properties and degradation of the scaffolds

**Figure 1.**
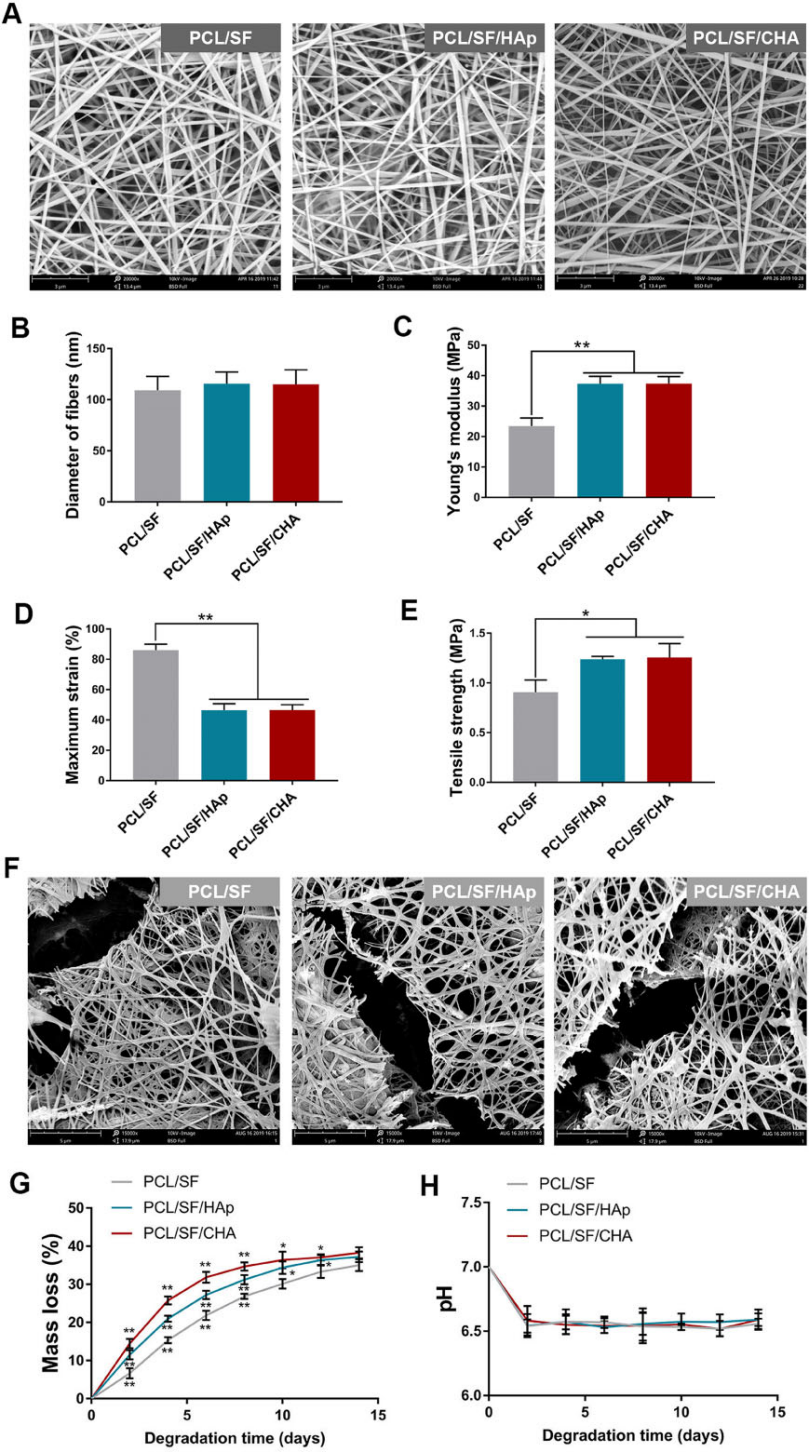
Surface morphology and mechanical strength of the scaffolds. (**A**) SEM of three kinds of scaffolds. The scale bar is 3 μm. (**B**–**E**) The average diameter of fibers (B), Young’s modulus (C), maximum strain (D) and tensile strength (E) of three kinds of scaffolds. (**F**) SEM images of the scaffolds after 4 days of degradation. The scale bar is 3 μm. (**G**) Mass loss of the scaffolds after 2, 4, 6, 8, 10, 12 and 14 days of degradation. (**H**) Change in pH of the incubation medium solution of the scaffolds after 2, 4, 6, 8, 10, 12 and 14 days of degradation. The *P* values represented by *P <* *0.05; **P <* *0.01; ***P <* *0.001.

The SEM analysis revealed the round and homogeneous morphology of HAp and CHA nanoparticles with a smaller size of CHA particles. The absorption peaks at 1415.22 cm^−1^, 1547.84 cm^−1^ and 1548.48 cm^−1^ indicated the presence of ${\text{CO}}_{3}^{2}$^–^ in CHA compared with HAp (Supplementary Fig. S1). Smooth and uniform nanofibers with a diameter of about 100 nm as well as porous structures were observed in each scaffold (Fig. 1A and B). In comparison to PCL/SF scaffold, the PCL/SF/HAp and PCL/SF/CHA scaffolds were found to possess higher Young’s modulus and tensile strength but lower maximum strain. However, no significant differences were found between PCL/SF/HAp and PCL/SF/CHA scaffolds in terms of Young’s modulus, tensile strength and maximum strain (Fig. 1C–E).

All scaffolds were fractured and broken in the aliphatic enzyme solution (Fig. 1F). Among the three types of scaffolds after 10 days of degradation, the PCL/SF/CHA scaffold was found to lose the most mass (more than 30%), whereas the PCL/SF scaffold lost the least amount (Fig. 1G). However, the weight loss ratio of the three types of scaffolds gradually approached after 14 days of degradation. No significant differences were found among the three groups in terms of the medium solution pH after 14 days of degradation (Fig. 1H).

### The hydrophilic and biocompatibility of scaffolds

**Figure 2.**
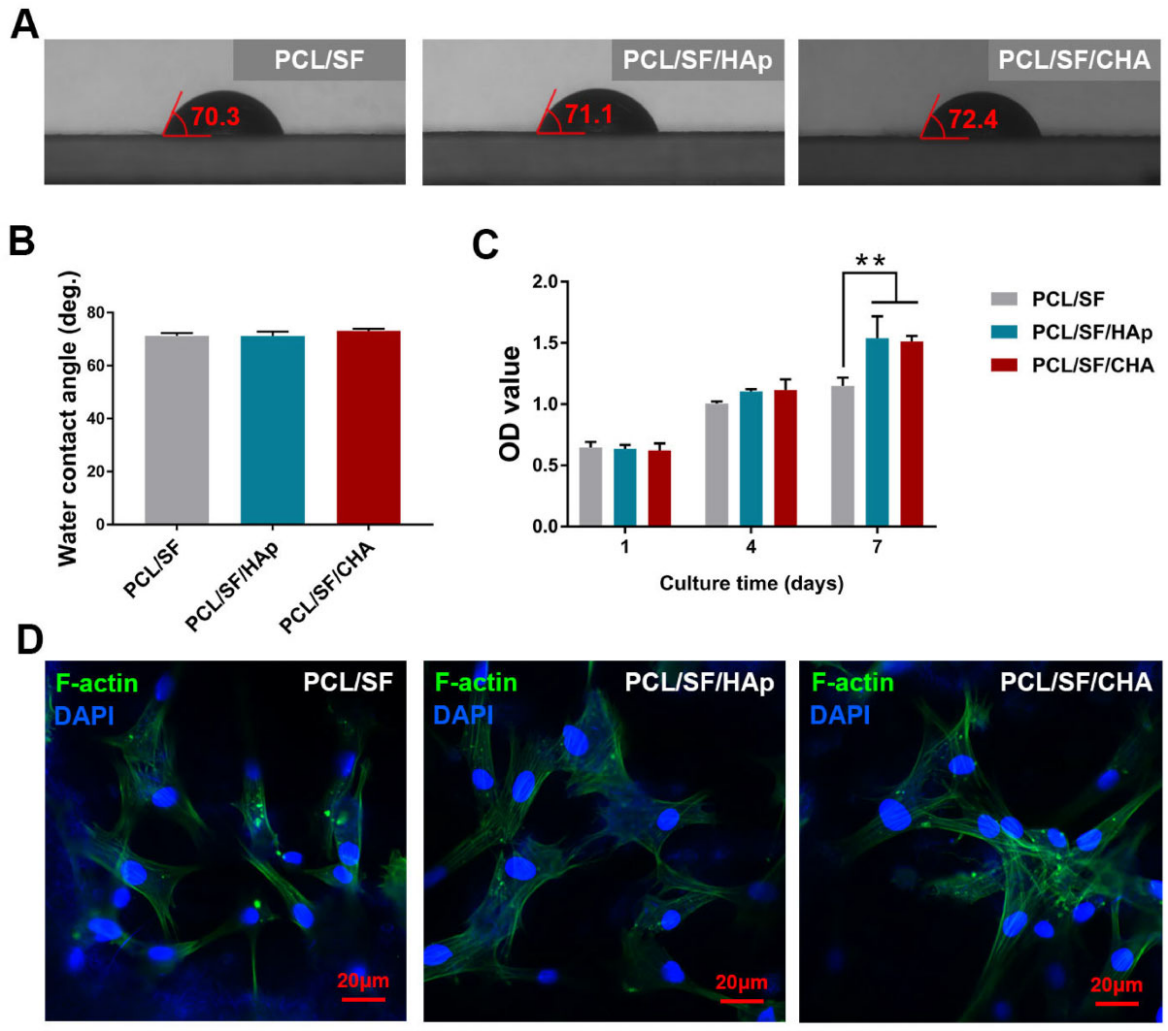
The hydrophilic and biocompatibility of scaffolds. (**A**) Images of the WCA of all scaffolds. (**B**) Quantitative analysis of the WCA of all scaffolds. (**C**) Cell proliferation of BMSCs cultured on scaffolds assessed by CCK8 assay. (**D**) The morphology of adhesive BMSCs cultured on scaffolds. The scale bar is about 20 μm and *P* values represented by *P <* *0.05; **P <* *0.01; ***P <* *0.001.

The water contact angle (WCA) of all scaffolds was around 70° (Fig. 2A and B). BMSCs proliferated well over time in all groups after 1, 4 and 7 days of cell culture. The proliferation of PCL/SF/HAp and PCL/SF/CHA groups was better than that of the PCL/SF group after 7 days of culture (Fig. 2C). The cells cultured on all scaffolds showed stretched or spindle morphology with apparent pseudopodia (Fig. 2D).

### The *in vivo* bone regeneration properties of scaffolds

**Figure 3.**
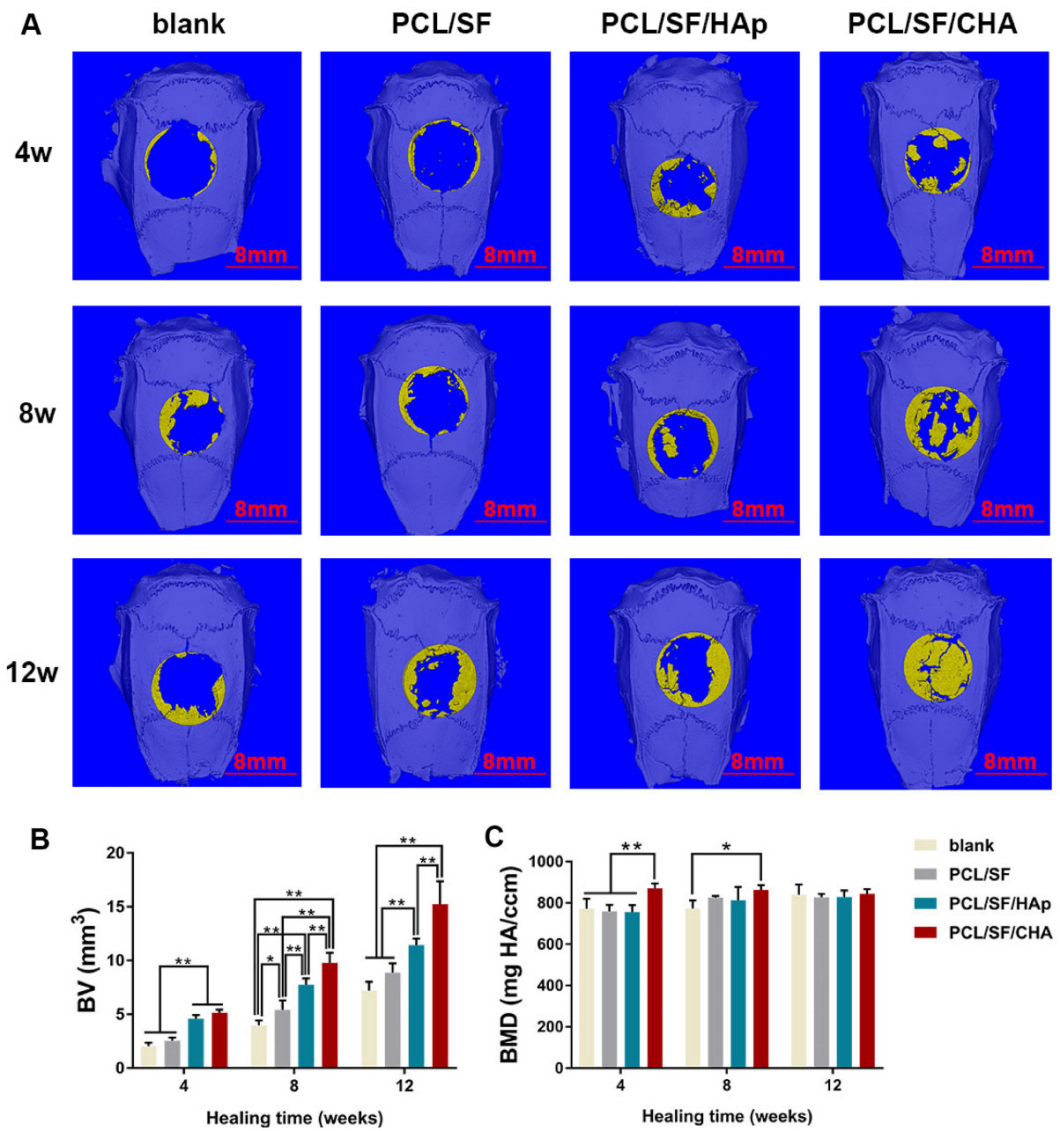
Effects of bone regeneration after 4, 8 and 12 weeks of surgery (*n* = 5). (**A**) Micro-CT images of blank, PCL/SF, PCL/SF/HAp and PCL/SF/CHA groups after 3D reconstruction. Yellow represents new bone formation. The scale bar = 8 mm. (**B**) Bone volume (BV) changes during bone regeneration. (**C**) Bone mineral density (BMD) of four groups during bone regeneration. The *P* values represented by *P <* *0.05; **P <* *0.01; ***P <* *0.001.

**Figure 4.**
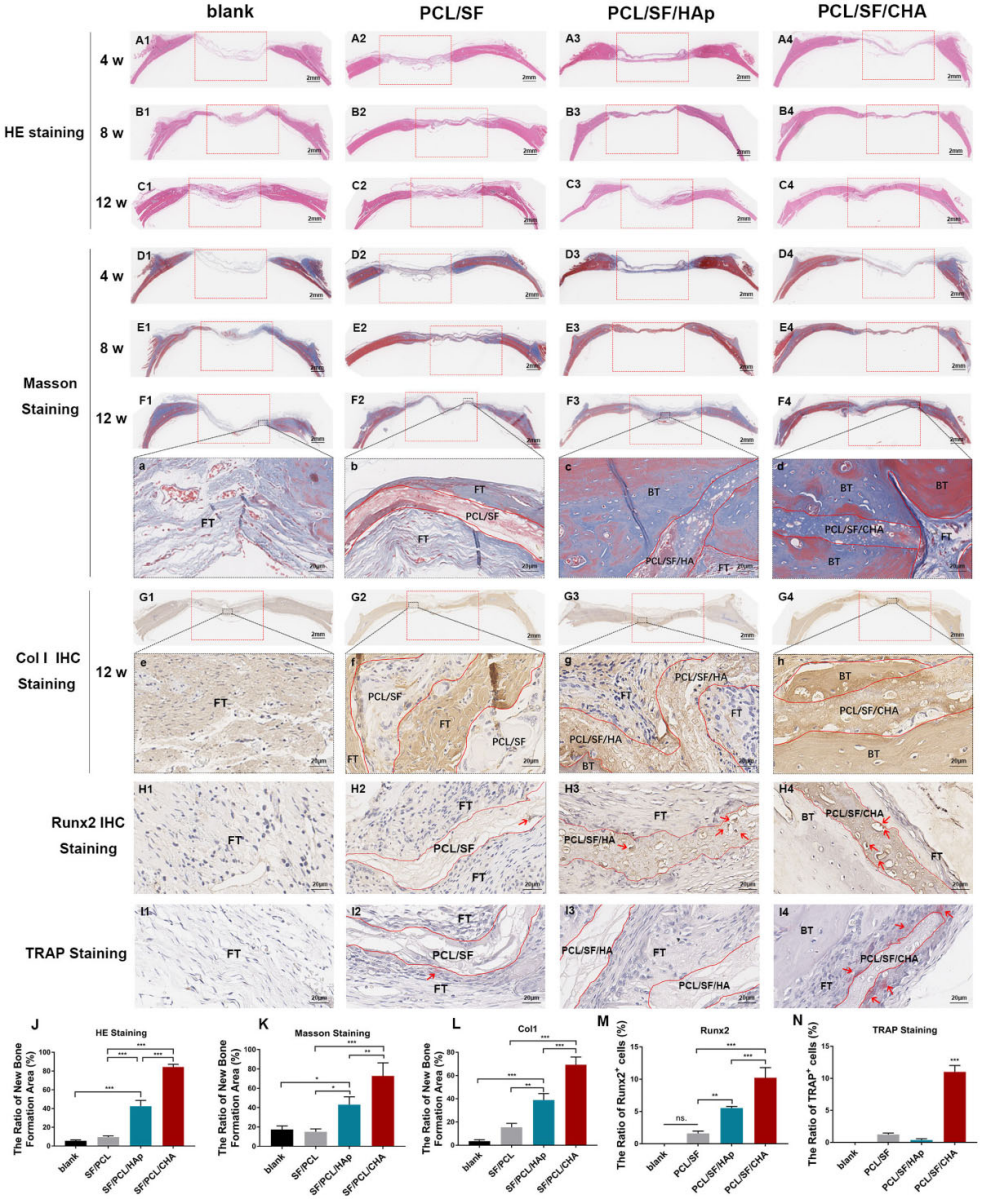
Histological staining of new bone regeneration (*n* = 5). (**A1**–**C4**): HE staining of the skull defect healing at 4 weeks (A1–A4), 8 weeks (B1–B4) and 12 weeks (C1–C4). (**D1**–**F4**, a–d): Masson staining of the skull defect healing at 4 weeks (D1–D4), 8 weeks (E1–E4) and 12 weeks (F1–F4, a-d). (**G1**–**G4**, e–h) Col I IHC staining of the skull defect healing at 12 weeks. (**H1**–**H4**): Runx2 IHC staining of the skull defect healing at 12 weeks. (**I1**–**I4**): TRAP staining of the skull defect healing at 12 weeks. (**J**–**N**): Quantitative analysis of HE (J), Masson (K), Col1 IHC (L), Runx2 IHC (M) and TRAP staining (N) at 12 weeks (A1–F4, G1–G4). The extent of the bone defects is shown by the width of the red boxes. The scale bar = 2 mm. (a–h, H1–I4) the scale bar = 20 μm. BT, bone tissue; FT, fibrous tissue; PCL/SF, PCL/SF scaffold; PCL/SF/HA, PCL/SF/HA scaffold; PCL/SF/CHA, PCL/SF/CHA scaffold. The dotted rectangle: scope of skull defect. The dotted curve: boundary of scaffolds. The *P* values represented by *P <* *0.05; **P <* *0.01; ***P <* *0.001.

The results of micro-CT and histological staining showed maximum new bone formation in the PCL/SF/CHA group (Figs 3A and B and 4A1–F4, J and K). In the PCL/SF/CHA group, the defect was almost filled with new bone after 12 weeks after surgery (Fig. 3A). Furthermore, quantification of the new bone volume showed better regeneration efficiency in the PCL/SF/CHA group (Fig. 3B). After 12 weeks, there were no significantly different among the four groups in terms of new bone mineral density (Fig. 3C).

Masson staining results showed that the surrounding fibrous connective tissue connected with or even grew into all scaffolds. No obvious dividing lines between scaffold and surrounding bone tissue were observed in PCL/SF/CHA group (Fig. 4a–d). The homogeneous bone matrix with embedded osteocytes in the defect area indicated the formation of mature bone structure (Fig. 4a–d). Moreover, the newly formed bone matrix was rich in well-organized Col I (Fig. 4e–h and L) and Runx2-positive cells (Fig. 4H1–H4 and M), which indicated excellent bone structure and more osteoblasts. The porous PCL/SF/CHA scaffold showed degeneration and was gradually replaced by bone tissue (Fig. 4a–d). Plenty of TRAP-positive cells gathered around PCL/SF/CHA scaffold indicated the good biodegradability (Fig. 4I1–I4 and N).

### The *in vitro* osteogenic properties of scaffolds

**Figure 5.**
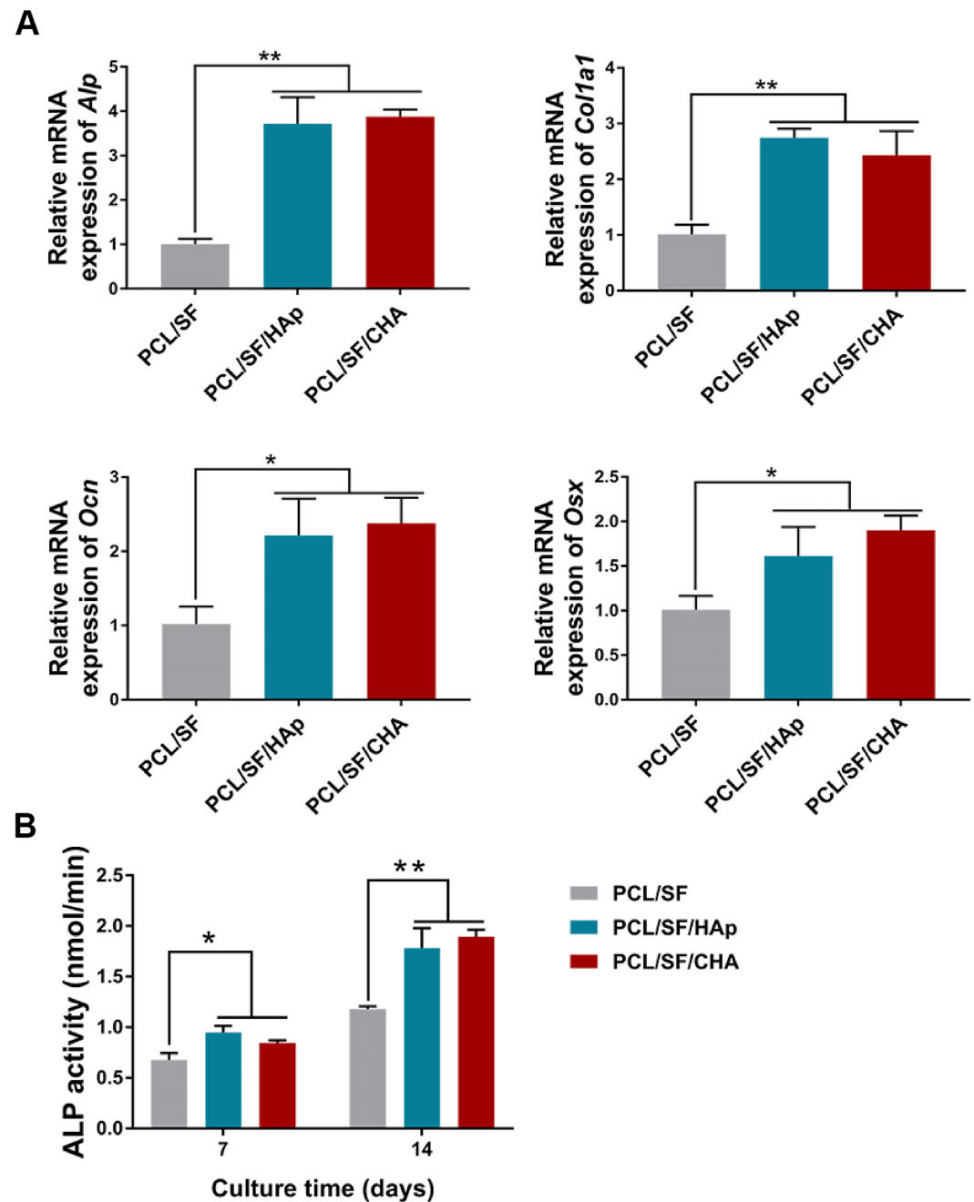
The osteogenic differentiation of BMSCs cultured on scaffolds. (**A**) The expression of *Alp, Col1a1, Ocn* and *Osx* of BMSCs after osteogenesis induction. (**B**) The ALP activity of BMSCs induced in osteogenesis induction medium for 7 and 14 days. The *P* values represented by *P <* *0.05; **P <* *0.01; ***P <* *0.001.

BMSCs cultured directly on PCL/SF/HAp and PCL/SF/CHA scaffolds had a higher expression of *Alp, Col1a1, Ocn* and *Osx* along with a higher ALP activity as compared to those cultured on PCL/SF scaffold. However, no significant difference was observed between the PCL/SF/HAp and PCL/SF/CHA groups (Fig. 5A and B).

### The osteoimmunological properties of PCL/SF/CHA scaffolds

**Figure 6.**
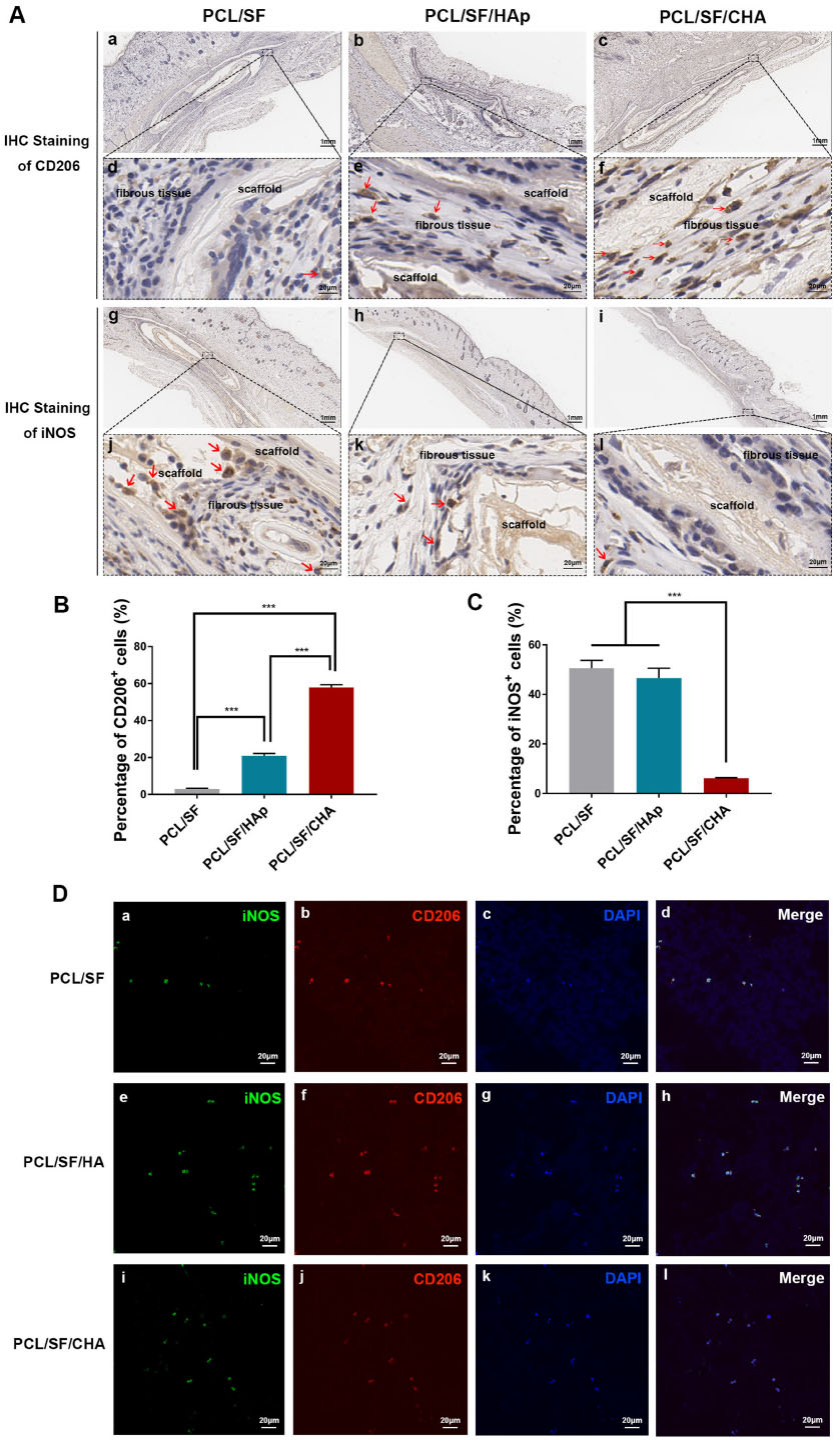
The polarization of macrophages around the scaffolds after subcutaneous implantation for 4 weeks. (**A**) The IHC staining against CD206 (**a**–**f**) and iNOS (**g**–**l**) of the macrophages around the scaffolds. (a–c, g–i) Scale bar = 1 mm. (d–f, j–l) Scale bar = 20 μm. (**B**) Quantitative analysis of CD206-positive cells. (**C**) Quantitative analysis of iNOS-positive cells. (**D**) The IF staining against CD206 and iNOS of RAW264.7 cultured on three kinds of scaffolds. The *P* values represented by *P <* *0.05; **P <* *0.01; ***P <* *0.001.

**Figure 7.**
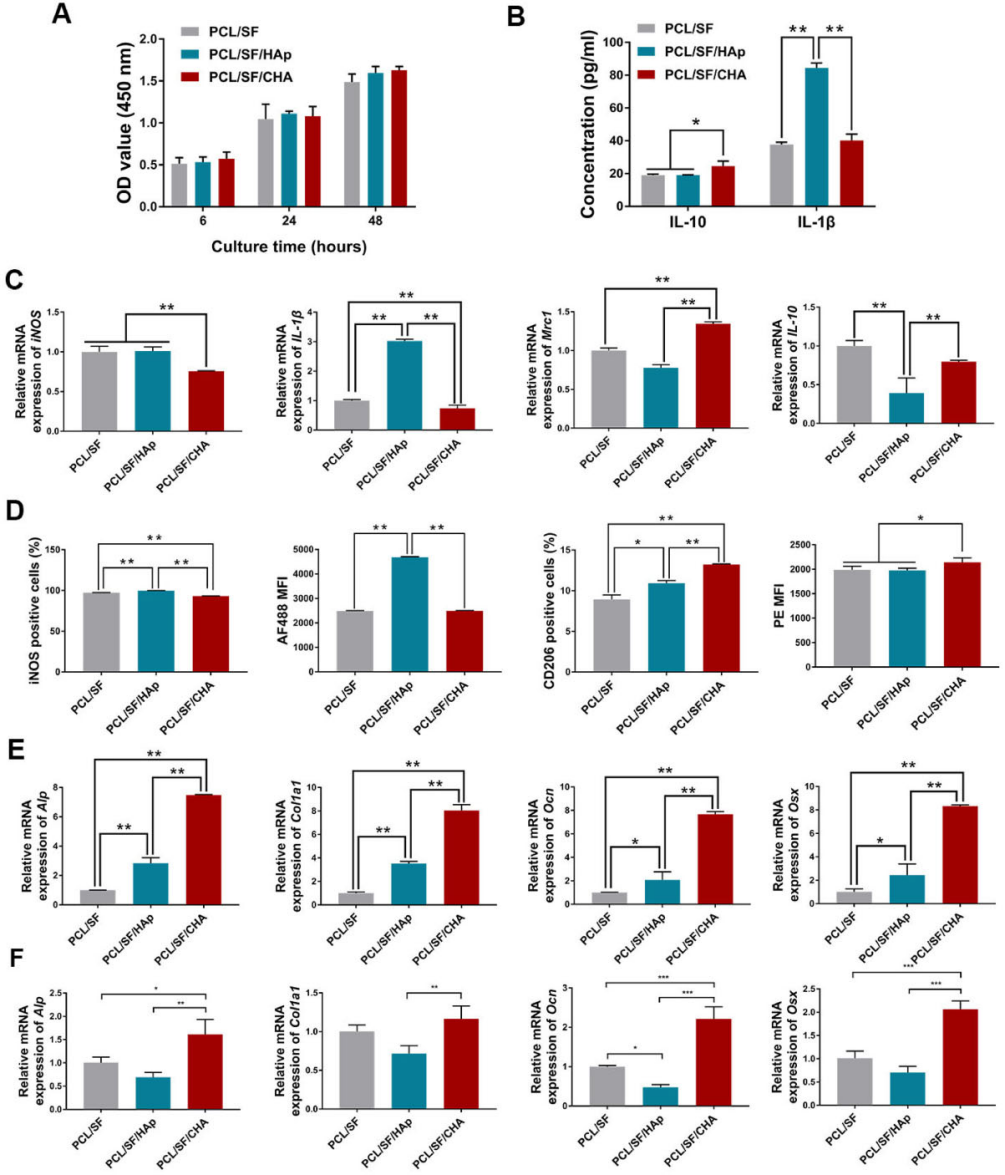
The PCL/SF/CHA scaffold promoted macrophage polarization to M2. (**A**) Cell proliferation of RAW 264.7 cells at 6, 24 and 48 h after culture on scaffolds, assessed by CCK8 assay. (**B**) ELISA assay of IL-10 and IL-1β released by RAW 264.7 cells cultured on the scaffolds. (**C**) The expression of *iNOS, IL-1β, Mrc1* and *IL-10* of RAW 264.7 cells cultured on the scaffolds. (**D**) The percentage and the MFI of iNOS- and CD206-positive cells of RAW 264.7 cells cultured on the scaffolds, analyzed by FCM. (**E**) The expression of *Alp, Col1a1, Ocn* and *Osx* of BMSCs cultured with RAW 264.7 cells on the scaffolds. (**F**) The expression of *Alp, Col1a1, Ocn* and *Osx* of BMSCs cultured in supernatant of RAW 264.7 cells on the scaffolds. The *P* values represented by *P <* *0.05; **P <* *0.01; ***P <* *0.001.

In contrast to the other groups, more CD206-positive (M2) macrophages and fewer iNOS-positive (M1) macrophages were observed around the PCL/SF/CHA scaffold after scaffolds were implanted subcutaneously for 4 weeks (Fig. 6A–C). RAW 264.7 cells cultured on different scaffolds showed the co-localization of CD206 and iNOS (Fig. 6D). The quantity of RAW 264.7 cells cultured on different scaffolds increased over time without significant differences among the three groups (Fig. 7A). Higher levels of IL-10 but lower levels of IL-1β released by RAW 264.7 cells in the PCL/SF/CHA group were observed as compared to the other 2 groups (Fig. 7B). In addition, RAW 264.7 cells in the PCL/SF/CHA group expressed higher levels of *Mrc1* and *IL-10* genes as well as lower levels of *iNOS* and *IL-1β* than did the other two groups (Fig. 7C). The mean fluorescence intensity (MFI) and the percent of iNOS-positive cells of RAW 264.7 cells in the PCL/SF/CHA group were lower, while those of CD206-positive cells were higher as compared to the other two groups (Fig. 7D).

Interestingly, the expression levels of *Alp, Col1a1, Ocn* and *Osx* of BMSCs cultured either with RAW 264.7 cells on the scaffolds or with supernatant of RAW 264.7 cells on the scaffolds were significantly higher in the PCL/SF/CHA group than in the other two groups (Fig. 7E and F), which was consistent with *in vivo* results.

### PCL/SF/CHA scaffold activated the JAK/STAT5 pathway and inhibited the AKT and NF-κB pathways in macrophages

**Figure 8.**
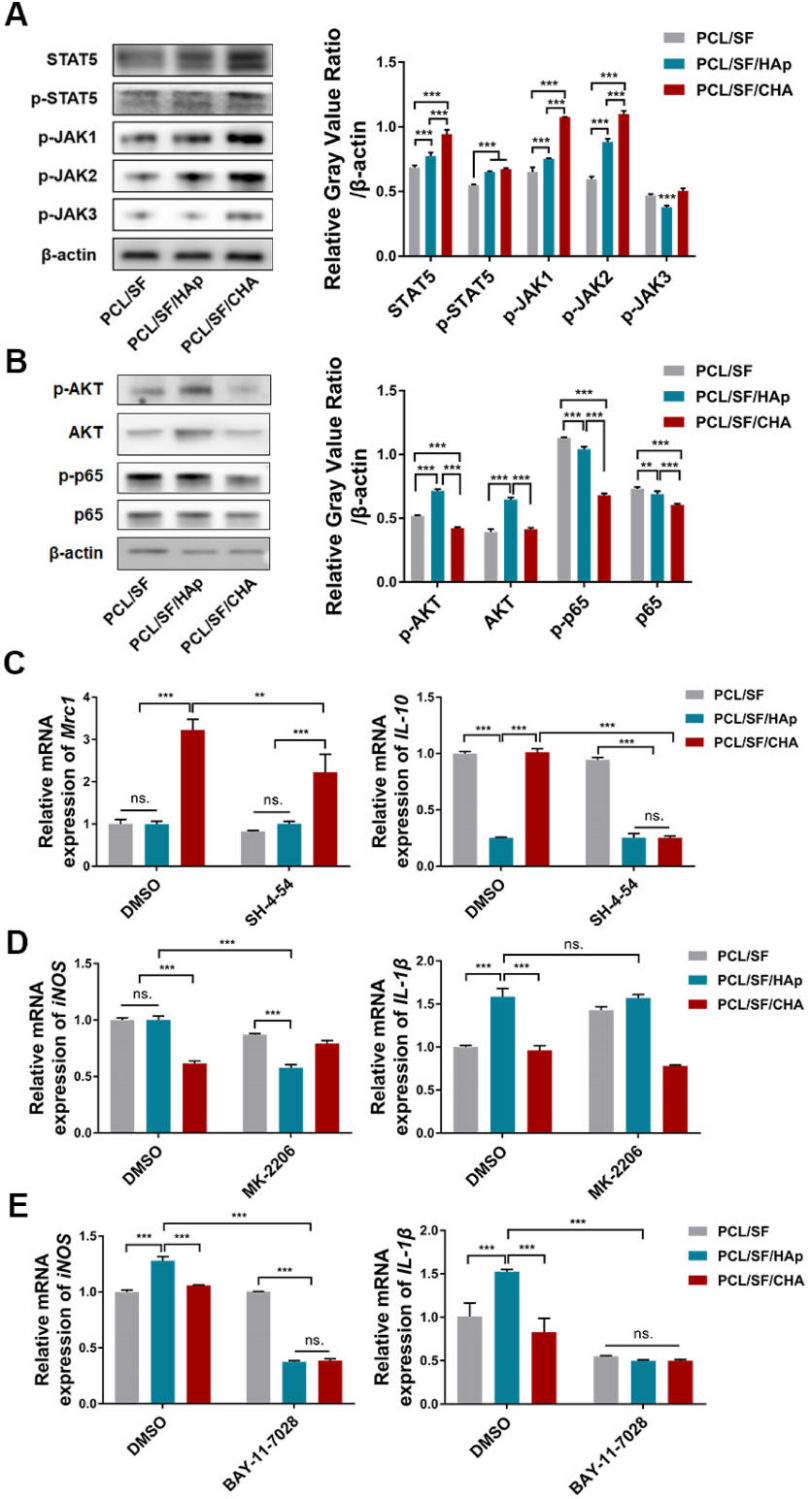
The PCL/SF/CHA scaffold activated the JAK/STAT5 pathway and inhibited the AKT and NF-κB pathways in macrophages. (**A** and **B**) Western blot of JAK/STAT5 pathway-associated proteins (A) and AKT and p65 pathway-associated proteins (B) in RAW 264.7 cells cultured on scaffolds. (**C**) The expression of *Mrc1* and *IL-10* in RAW 264.7 cells. RAW 264.7 cells were pretreated by JAK/STAT5 inhibitor SH-4-54 (10 μM) and then cultured on the scaffolds. (**D** and **E**) The expression of *iNOS* and *IL-1β* in RAW 264.7 cells. RAW 264.7 cells were pretreated by 10 μM AKT inhibitor MK2206 (D) or 10 μM NF-κB inhibitor Bay-11-7028 (E) and then cultured on the scaffolds. The *P* values represented by *P <* *0.05; **P <* *0.01; ***P <* *0.001.

Western blotting results showed that in the PCL/SF/CHA group, the protein levels of phosphorylated JAK, p-STAT5 and STAT5 were higher (Fig. 8A), whereas the protein levels of p-AKT and p-p65 were lower as compared to the other two groups (Fig. 8B). After the JAK/STAT5 pathway was blocked with specific inhibitor SH-4-54, the mRNA expression of *Mrc1* and *IL-10* in RAW 264.7 cells cultured on the PCL/SF/CHA scaffold was significantly suppressed (Fig. 8C). In both the PCL/SF/HAp and PCL/SF groups, inhibition of the AKT pathway caused less expression of only *iNOS* but not *IL-1β* (Fig. 8D). Significant suppressed expression of *iNOS* in PCL/SF/HAp group and *IL-1β* in both PCL/SF/HAp and PCL/SF groups following inhibition of NF-κB (Fig. 8E).

## Discussion

### The physical properties of PCL/SF/CHA scaffolds

Electrospinning is a simple and effective technique used to producing nanofibers [21]. By adjusting the parameters of electrostatic spinning apparatus, nanofibers of various shapes and diameters can be produced. Using electrospinning fiber membrane, bone matrix nanofibers can be simulated. The rough surface and certain dynamic compression strain of electrospinning fiber membrane can affect osteoblasts proliferation and differentiation [22, 23]. Additionally, porous electrospinning scaffolds can be loaded with proteins and drugs using some specific technologies [23]. In the past, researchers have developed electrospinning bone scaffolds for skull defects and alveolar bone defects regeneration [24, 25]. Electrospinning periosteum showed the capability of accelerating bone regeneration and promoting angiogenesis [26]. Nanofibrous membrane prepared using electrospinning technique showed the ability to improve new bone generation, which has also been applied in guided bone regeneration [27]. These studies indicate the wide application prospect of electrospinning bone graft.

PCL has been widely used as the raw material for electrospinning in bone tissue engineering due to its favorable mechanical and biodegradable properties [4]. As a naturally derived biocompatible polymer, SF is an attractive electrospinning biomaterial to improve the biocompatibility of PCL in tissue engineering [28]. The composition of ceramics is found to be comparable to that of natural bone, and it exhibits the desired mechanical properties. The incorporation of the HAp into polymer scaffolds has shown to improve strength and crack-resistance [29, 30]. By providing bioactive sites, HAp can improve the osteogenic performance of many scaffolds [8]. CHA consists of 7.4 (wt%) carbonate ions, therefore is more similar as the mineral portion of bone as compared to HAp [11]. Our results showed the excellent spinnability of the PCL/SF, PCL/SF/HAp and PCL/SF/CHA solutions. The synthesized microfibers were smooth and uniform without clusters of HAp or CHA. The addition of HAp or CHA increased the Young’s modulus and tensile strength of the PCL/SF scaffold. Moreover, the replacement of HAp by CHA did not reduce the mechanical properties of the scaffold.

On this basis, the PCL/SF/CHA scaffold presented more rapid degradation and higher pH of the degradation medium. The presence of carbonates in the CHA lattice can increase the solubility in acid medium compared with that of HAp, enhancing absorption by osteoclasts and promoting bone regeneration *in vitro* and *in vivo* [31]. In the process of bone tissue remodeling, bone is resorbed and subsequently regenerated. A CaP-based material is degraded by two mechanisms, one is the dissolution due to physicochemical solution, and the other is the resorption by osteoclasts [12]. However, the *in vitro* and *in vivo* solubility of HAp is found to be limited [32]. In native bone tissue, nanocrystals exists as carbonated HAp, whose solubility is higher than the solubility of HAp [11]. Due to the weaker bonds of Ca-CO_3_ than Ca-PO_4_, the present of carbonate could decrease crystallinity, leading to an increase in solubility [33, 34], which is coordinate with the morphology and XRD of CHA and HAp particles in our experiments. It has been proved that both nanocrystalinity and carbonate content promotes the resorption by osteoclasts [35–37]. In the process of the resorption of bone and bone substitution materials, first, osteoclasts attach to the bone surface by forming a sealing zone between the cell membrane and the mineralized matrix. Second, osteoclasts release protons into the sealing zone of which has a pH of around 4 [10, 38–40]. Third, CHA nanoparticles can be taken up by osteoclasts through endocytosis and relate mechanism, which proceed in the form of endosomes and fuse with lysosomes.

Degradation rate can also be modified by changing the superficial area or adding a component that degrades faster [41, 42]. During degradation, the particles of HAp and CHA were free from nanofibers, which increased the superficial area of fibers. The smaller CHA particles dissociated more quickly and led to the more rapid degradation of the PCL/SF/CHA scaffold. The degradation products of PCL were acidic due to the ester chain degradation [19, 43], so the pH of the medium in each group decreased during degradation.

### The biocompatibility and bone regeneration properties of PCL/SF/CHA scaffolds

Despite PCL being inert, hydrophobic and lacking biological recognition sites, the addition of SF improved the hydrophilic properties of scaffolds, which led to WCAs < 90° in all scaffolds. The BMSCs adhered and were fully stretched on all scaffolds, indicating that the surface features of the scaffolds allowed cell growth. The stable proliferation of BMSCs also showed the good biocompatibility of the scaffolds.

PCL/SF/CHA scaffolds also promoted bone regeneration excellently. We investigated the bone regeneration of CSD models with scaffolds over a 12-week healing period because the healing time for clinical bone defects is about 3–6 months. The stem cells and blood vessels within the fibrous connective tissue infiltrated the PCL/SF/CHA scaffold. The scaffold was embedded in the surrounding new bone without boundary and resembles the bone matrix. After 12-week healing, the mineralized new bone almost filled the defect area in the PCL/SF/CHA group. These results indicated the coordination and balance between bone formation and scaffold degradation. The new bone consists of bone matrix rich in well-organized Col I and the embedded osteocytes, forming an alive and compressible structure.

Bone matrix formation and bone mineralization directly depend on the differentiation of stem cells and osteoblasts. HAp and CHA are rich in Ca^2+^ and ${\text{PO}}_{4}^{3}$^–^ ions, which provide calcium and phosphorus for bone formation. The BMSCs cultured directly on PCL/SF/HAp and PCL/SF/CHA scaffolds showed higher osteogenic-associated gene expression and ALP activity than PCL/SF group. However, the differentiation of BMSCs in the PCL/SF/HAp and PCL/SF/CHA groups showed no differences, which is inconsistent with the *in vivo* study of bone formation. The results left many questions unanswered and prompted us to determine the underlying reasons.

### PCL/SF/CHA scaffolds promoted M2-type macrophages polarization

An inflammatory response mediated by macrophages happens in the early stage of bone repair promoted by biomaterials [44]. Macrophages are involved in nearly every biological process of bone formation after biomaterial implantation, including the initial immune process [45], mesenchymal stem cell recruitment [46] and osteoblast differentiation and angiogenesis [17]. The interaction between biomaterial scaffolds and macrophages brought new insight into the mechanisms involved in bone formation. In a study by Igeta and coworkers [13], it was shown that the secretion levels of inflammatory cytokines by RAW 264.7 macrophage cells spreading on the nCHA disk decreased when compared to nHAp group. In scaffolds containing nCHA instead of nHAp, macrophages may be polarized in a way that promotes the osteogenic differentiation of mesenchymal stem cells and other pre-osteogenic cells. In this study, the images of HE and Masson staining showed both blood vessels and fibrous tissues, where may be the source of macrophages grew into the PCL/SF/CHA scaffold and the new bone. IHC staining and *in vitro* study indicated that the PCL/SF/CHA scaffold has a superior effect on macrophage polarization toward the M2, which secrete pro-wound-healing cytokines and growth factors including VEGF, TGF-*β* and IFG-1 to promote immunoregulation and tissue repair [16]. With the culture of RAW 264.7 cells on PCL/SF/CHA scaffolds, BMSCs showed better osteogenic differentiation than other groups, indicating macrophages play an intermediary role in bone formation induced by the PCL/SF/CHA scaffold. The addition of RAW 264.7 may provide direct effect through the contact between cells, or indirect effect through secreting cytokines. Therefore, we added the supernatant of the RAW 264.7 cultured on scaffolds, which contains cytokines secreted by RAW 264.7 but no cells, into the osteogenic induction medium of BMSCs. The results showed consistent with that of co-culture system. Therefore, we have reason to believe macrophages played an immunomodulatory role by secreting cytokines to create a pro-osteogenesis microenvironment in the process of bone regeneration after scaffolds implantation [13]. Therefore, we speculate that the CHA is responsible for the immunomodulation effect of PCL/SF/CHA on macrophages.

Multiple inflammation-associated pathways induce macrophage polarization. The NF-κB, AKT and JAK/STAT pathways are three classic pathways regulating the polarization of macrophages [44]. The activation of canonical NF-κB is considered critical in the M1 program [47], while the JAK/STAT pathway has been proven to mediate M2 polarization [48]. The role of the AKT pathway in macrophage polarization remains controversial, due to the variation in the Akt protein isoforms [49, 50]. Recently, a nonclassical TLR-mediated signaling pathway regulating macrophage phenotype has been found [51]. Phosphorylated STAT5 can suppress the transcription of inflammatory cytokines and promote expression of *Arg1*, a gene associated with M2 macrophage polarization and tissue repair [51]. In this study, we found that the JAK/STAT5 pathway was activated but that the AKT and NF-κB pathways were inhibited in RAW 264.7 cells cultured on the PCL/SF/CHA scaffold. The inhibition of the JAK/STAT5 pathway attenuated the expression of M2 markers, whereas the inhibition of the AKT and NF-κB pathways caused less expression of M1-associated genes.

The shape, surface morphology, each composition, degradation products or microenvironment after degradation of the scaffolds such as PH could be the cause of the M2-type macrophages polarization. The upstream receptors of JAK/STAT pathway include growth hormones receptors, IFN receptors, cytokine receptors, G-protein-coupled receptors and receptor tyrosine kinase. Considering that various factors of the scaffolds influence the biological process in macrophages, the target receptors for PCL/SF/CHA scaffolds maybe also complex. In our future studies, we should explore the specific factors that affect macrophages polarization in response to PCL/SF/CHA scaffolds and the receptors that binds to the scaffold to provide more theoretical basis for the improvement of the scaffolds.

## Conclusions

The PCL/SF/CHA scaffold exhibited preferable mechanical, degradation and bone-formation-promoting properties. The PCL/SF/CHA scaffold could regulate macrophage polarization and create a pro-osteogenic microenvironment. CHA has great potential to replace HAp in the arsenal of biomaterials for bone tissue engineering.

## Supplementary data

Supplementary data are available at *REGBIO* online.

## Funding

This work was supported by funds from the National Natural Science Foundation of China (82071156 to K.Z. and 81901025 to X.J.), from the Guangdong Basic and Applied Basic Research Foundation (2019A1515011326 to X.J.) and by the Fundamental Research Funds for the Central Universities (19ykpy82 to X.J.).

*Conflicts of interest statement*. The authors declare that there is no conflict of interest regarding the publication of this article. This original research is free of conflict of interest.

## Data availability statement

The raw/processed data that support the findings of this study are available from the corresponding authors, XW., Y.J. and K. Z., upon reasonable request.

## Supplementary Material

rbac035_Supplementary_Data
