# Supplementary material for: The polycaprolactone/silk fibroin/carbonate hydroxyapatite electrospun scaffold promotes bone reconstruction by regulating the polarization of macrophages
Source: Regen Biomater. 2022 Jun 11;9:rbac035. doi: 10.1093/rb/rbac035 (PMC9255275; doi:10.1093/rb/rbac035)
Supplement: rbac035_Supplementary_Data [file rbac035_supplementary_data.docx]

**Supplementary Material**

The polycaprolactone/silk fibroin/carbonate hydroxyapatite electrospun scaffold promotes bone reconstruction by regulating the polarization of macrophages

Xiaoshi Jia^a,b,#^, Jing Zhou^a,b,#^, Jinqiu Ning^a,b,#^, Maoquan Li^a,b^, Yitong Yao^a,b^, Xiaodong Wang^a,b,*^, Yutao Jian^a,b,*^, Ke Zhao^a,b,*^

*^a^Guanghua School of Stomatology, Hospital of Stomatology,* *Sun Yat-sen University, 56 Lingyuan West Road, Guangzhou, Guangdong 510055, China*

*^b^Guangdong Provincial Key Laboratory of Stomatology, Guangzhou, China*

**1. Materials and methods**

*1.1. Materials*

Stoichiometric pure hydroxyapatite, Ca(NO_3_)_2_·4H_2_O, (NH_4_)_2_HPO_4_, NH_4_HCO_3_, ammonia solution and ethyl alcohol were supplied by the Aladdin Company (China). Poly(ε-caprolactone) PCL (MW = 80,000) was supplied by the Sigma-Aldrich Chemical Corporation (USA). *Bombyx mori* cocoons were bought from Northwest Silkworm Base (China). Hexafluoroisopropanol (HFIP, 99.5%) was purchased from Shanghai Macklin Biochemical Co. (China). All chemicals were of analytical grade and utilized without further purification.

*1.2. CHA preparation*

Carbonate hydroxyapatite powders were prepared by an aqueous precipitation reaction as described [1], with some adjustments. Briefly, a solution of Ca(NO_3_)_2_·4H_2_O and a mixed solution of (NH_4_)_2_HPO_4_ and NH_4_HCO_3_ with a molar ratio of CO_3_^2-^ to PO_4_^3-^ of 0.5:1 were prepared with specific concentrations, both adjusted previously to the desired pH with an ammonia solution. In the process of mixing, a functionalization agent was added to protect the particles from aggregating too soon [2]. The obtained precipitate was maintained at 180 ℃ for 8 h, then cooled, vacuum-filtered, washed with distilled water and ethyl alcohol, and vacuum-freeze-dried.

*1.3. Preparation of* *regenerated* Bombyx mori *silk fibroin (BMSF)*

Regenerated *Bombyx mori* silk fibroin was prepared as described previously [3]. Briefly, *Bombyx mori* cocoons were boiled for 30 min in a 0.02 M Na_2_CO_3_ solution and rinsed thoroughly with distilled water for 1 h to remove the sericin proteins, then dried overnight. The degummed silk fibroin was then dissolved in a 9.3 M LiBr solution at 60 ℃ for 4 h, yielding a 20% (w/v) solution. This solution was dialyzed against distilled water with a dialysis tube (MWCO 14,000) for 48 h before being centrifuged twice at 9000 rpm and 4 ℃ for 20 min each time for the removal of possible impurities. Finally, the aqueous fibroin solution was lyophilized to obtain dry regenerated SF plates.

*1.4.* *Characterization of HAp and CHA powders*

The X-ray diffraction (XRD) patterns of HAp and CHA powders were obtained by means of a polycrystal X-ray diffractometer (SmartLab SE, Rigaku, Japan) operating with a CuK_α_ (λ = 0.15406 nm) radiation source at 40 kV and 40 mA. Diffraction patterns were collected from 5° to 90° with a step size of 0.0131303°, and 15.045 s per step was used. The Fourier transform infrared spectroscopy (FTIR) spectra of the powders were obtained by means of an FTIR spectrometer (VERTEX 70, Bruker, Germany) for study of the carbonate substitutions according to the KBr standard. The HAp and CHA powders were sputter-coated with gold and observed under a SEM (SU8200, Hitachi, Japan) at 10 kV accelerating voltage (Fig. S).


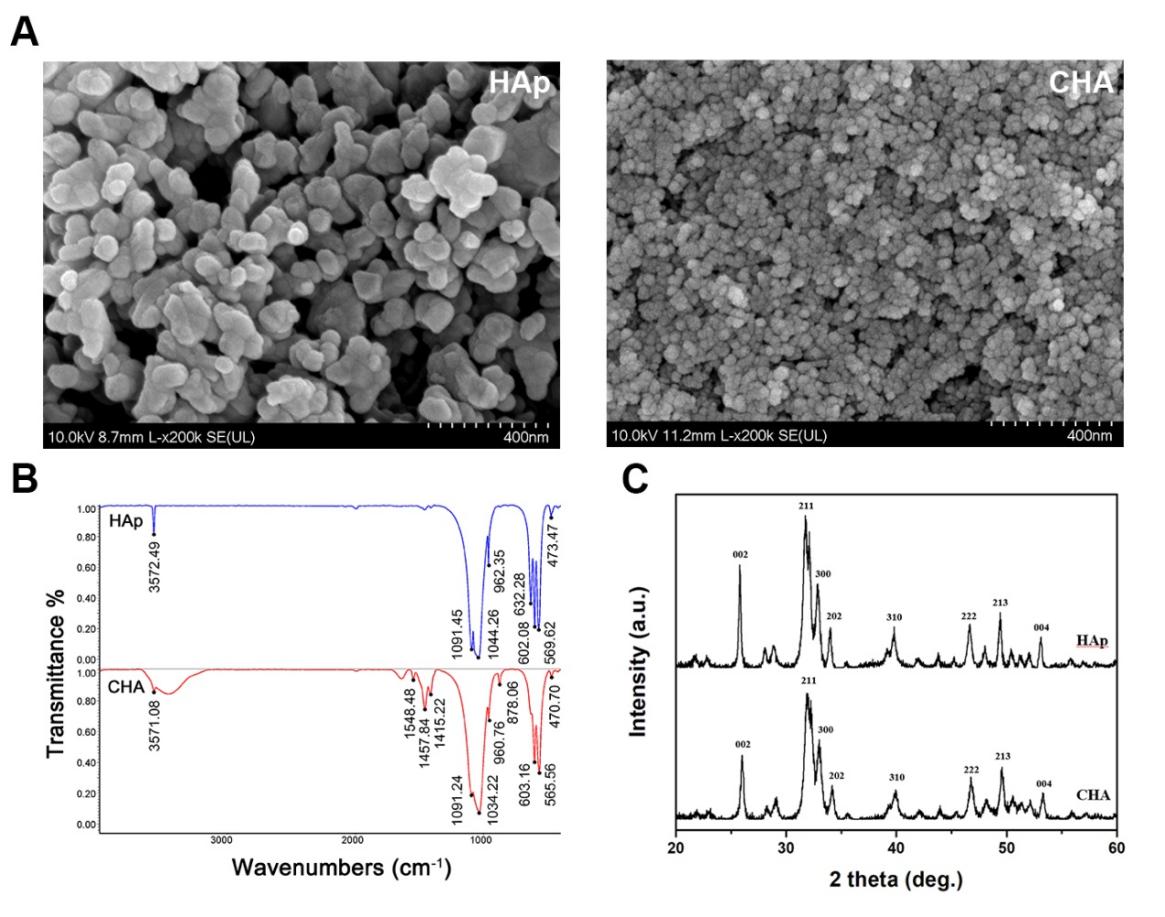


**Fig. S.** Characterization of HAp and CHA powders. (A) SEM images of HAp and CHA powders. The shape of Hap and CHA particles are spherical with diameters around 80 nm and 40nm separately. (B) The FTIR spectra of HAp and CHA powders. (C) XRD of HAp and CHA powders.

**References**

[1] D. Tang, R.S. Tare, L.Y. Yang, D.F. Williams, K.L. Ou, R.O.C. Oreffo, Biofabrication of bone tissue: approaches, challenges and translation for bone regeneration, Biomaterials 83 (2016) 363-382.

[2] D.Q. Xiao, J.W. Zhang, C.D. Zhang, D. Barbieri, H.P. Yuan, L. Moroni, G. Feng, The role of calcium phosphate surface structure in osteogenesis and the mechanisms involved, Acta Biomater. 106 (2020) 22-33.

[3] J.T. Zhang, W.Z. Liu, V. Schnitzler, F. Tancret, J.M. Bouler, Calcium phosphate cements for bone substitution: chemistry, handling and mechanical properties, Acta Biomater. 10 (2014) 1035-1049.
